# Rheological and the Fresh State Properties of Alkali-Activated Mortars by Blast Furnace Slag

**DOI:** 10.3390/ma14082069

**Published:** 2021-04-20

**Authors:** Markssuel Teixeira Marvila, Afonso Rangel Garcez de Azevedo, Paulo Ricardo de Matos, Sérgio Neves Monteiro, Carlos Maurício Fontes Vieira

**Affiliations:** 1 LAMAV-Advanced Materials Laboratory, UENF-State University of the Northern Rio de Janeiro, Av. Alberto Lamego, 2000, 28013-602 Campos dos Goytacazes, Brazil or m.marvila@ucam-campos.br (M.T.M.); or snevesmonteiro@gmail.com (S.N.M.); vieira@uenf.br (C.M.F.V.); 2 LECIV-Civil Engineering Laboratory, UENF-State University of the Northern Rio de Janeiro, Av. Alberto Lamego, 2000, 28013-602 Campos dos Goytacazes, Brazil; 3 Department of Civil Engineering, UFSC–Federal University of Santa Catarina, Rua João Pio Duarte Silva, 205, 88040-900 Florianópolis, Brazil; paulo.matos@ufsm.br or; 4 Coordenadoria Acadêmica, UFSM–Federal University of Santa Maria, Rodovia Taufik Germano, 3013, 96503-205 Cachoeira do Sul, Brazil; 5 Department of Materials Science, IME—Military Institute of Engineering, Square General Tibúrcio, 80, 22290-270 Rio de Janeiro, Brazil

**Keywords:** rheological, blast furnace slag, alkaline activation

## Abstract

The fresh and rheological properties of alkali mortars activated by blast furnace slag (BFS) were investigated. Consistency tests, squeeze flow, dropping ball, mass density in the hardened state, incorporated air, and water retention were performed. Mortars were produced with the ratio 1:2:0.45 (binder:sand:water), using not only ordinary Portland cement for control but also BFS, varying the sodium content of the activated alkali mortars from 2.5 to 15%. The results obtained permitted understanding that mortars containing 2.5 to 7.5% sodium present a rheological behavior similar to cementitious mortars by the Bingham model. In turn, the activated alkali mortars containing 10 to 15% sodium showed a very significant change in the properties of dynamic viscosity, which is associated with a change in the type of model, starting to behave similar to the Herschel–Bulkley model. Evaluating the properties of incorporated air and water retention, it appears that mortars containing 12.5% and 15% sodium do not have compatible properties, which is related to the occupation of sodium ions in the interstices of the material. Thus, it is concluded that the techniques used were consistent in the rheological characterization of activated alkali mortars.

## 1. Introduction

Alkali-activated materials are produced through the alkaline activation of two classes of binders, which are subdivided according to their chemical composition: (i) the rich in calcium [1] and (ii) the rich in aluminosilicates, which make up the class of geopolymeric materials [2,3]. Although there is a large amount of published research within the latter geopolymers, there is a lack of studies related to alkaline-activated calcium-rich binders, such as blast furnace slag (BFS) mortars [4].

BFS is a supplementary binder used in partial substitutions of Portland cement, with great possibility of application in an isolated way. In the presence of cement, BFS exhibits binding power due to the presence of Ca(OH)_2_ from Portlandite, which causes the material to activate its hydration reaction [5]. Research works have proven the use of BFS with other binders, as mentioned below: Karthirvel et al. [6] studied the application of fly ash and BFS activated in substitution to Portland cement concrete, obtaining results of compressive strength greater than 60 MPa at 28 days. Abdulkareem et al. [7] investigated the feasibility of using residual glass powder as a partial precursor in fly ash and granulated BFS based on alkali-activated mortars cured in ambient conditions. Zhang et al. [8] studied the use of activated alkali mortars containing BFS and fly ash activated with sea water and containing aggregates of coral sand. Ahmad et al. [9] evaluated the durability properties of different compositions of alkaline-activated mortars using Portland cement, active silica, fly ash, BFS, magnesia, and metakaolin as binders. In all the research highlighted, the authors proved that the use of BFS is feasible, even if in conjunction with other binders.

It has been observed that despite the great potential of applying BFS as an isolated binder, there are only limited studies that carry out this type of research. This scenario is even more scarce when one takes into account the behavior of this material in the fresh state and its rheological properties. The rheological parameters of alkaline-activated mortars are extremely important. Indeed, these materials are not very reactive in the presence of water, requiring the use of alkaline solutions, based on NaOH, KOH, and other components [10,11]. This is the case of BFS.

The main solutions used are the NaOH base, which considerably alter the viscosity of mortars in the fresh state. This is because the sodium content in the material, even if dissolved, forms an additional barrier and provides more friction between the grains of the fresh mortar, compromising the flow of the material and consequently its workability [12,13]. On the other hand, it is known that the sodium content is a factor that directly affects the mechanical behavior of the alkaline-activated mortars. In fact, resistant compounds are formed due to the introduction of sodium in the interstices of the C-A-S-H (calcium aluminosilicate hydrated) networks [14]. Thus, in this research, it was decided to evaluate the effect of sodium content on the rheological properties and the fresh state of BFS-based mortars, as this is a major factor in the behavior of alkaline-activated materials.

As such, the objective of this article is to evaluate the rheological properties and the fresh state of mortars alkali-activated by BFS. The results will be compared with these of a cementitious mortar, whose behavior is already known in the literature. The behavior of mortars in the fresh state is of paramount importance to predict properties of the material in terms of workability [15,16]. Therefore, the main theoretical aspects of each evaluated parameter will be highlighted in the sequence of the text.

The first property that will be discussed is the mass density in the fresh state and the content of incorporated air. These two properties are closely related, since to calculate the incorporated air, it is necessary to know the real density (in the fresh state) and the theoretical density calculated based on the components that make up the mortar according to NBR 13278 [17]. The percentage difference between the actual density and the theoretical density is attributed to the incorporated air.

Some authors [18,19,20] describe that the air incorporated in mortars is an indirect parameter of workability, since the greater the incorporated air, the lower the internal friction between the particles that make up the mortar, improving the fluidity and workability of the mortar. From this point of view, the incorporated air acts as a kind of lubricant for the grains of the dry materials (cement, lime, sand, and metakaolin) that make up the material. However, the same research group [21,22,23] emphasizes that if the content of incorporated air is very high, the resistance and durability of the mortar in the hardened state will be compromised, because the greater the amount of air inside the material, the greater will be the amount of pores or voids when hardening occurs. In addition, the incorporated air content is related to other parameters, such as the sand and contents as well as binders and the presence or absence of chemical additives. Therefore, this property must be evaluated with caution, which is mainly due to the absence of limit values in international technical standards, although some authors recommend a useful range between 7% and 17% of incorporated air [24].

In the particular case of alkali-activated mortars, some authors point out that the excess of incorporated air is harmful to the mortars for another reason: the internal air bubbles are preferred places for the accumulation of sodium, potassium, or any other alkali metal involved in the alkaline activation reaction [25]. Alkaline ions do not chemically bind to the gels formed in the alkaline activation reaction, locating themselves in the interstices of the material. Thus, when an excess of incorporated air occurs, the alkaline ions preferentially locate in these places. When the mortar hardens, they react with atmospheric air, causing efflorescence, reducing the resistance of the material, and causing other material pathologies [26,27].

Regarding the property of mass density in the fresh state, in addition to being used to calculate the content of incorporated air, some authors consider the analysis of this property important in another aspect. When the mortar is cast horizontally by workers, either for wall cladding or for reinforcing damaged structures, the density of the material is an important factor because if the mortar is too heavy, even if it presents good adhesion parameters, it will not remain stuck to the substrate due to gravity [18,22]. In the vertical downward release, this property is not important. However, in the vertical upward release, either by coating or reinforcement of the ceiling, excessive values of mass density in the fresh state can compromise the performance of the mortar. The mass density is also related to other relevant information on the mortar behavior, such as the difference in relation to the textural analysis of the substrate, the position of the substrate, and the fluidity or not of the mortar. International standards do not define limit values for density, making it difficult to analyze this parameter. However, authors report density ranging from 1.80 to 2.10 g/cm³ for cementitious mortars [19,21,28,29] and activated alkali [27,30,31] with satisfactory adhesion values. Therefore, these values might be used for comparison.

Regarding the water retention, research on mortar reported that it is an important property directly related to mechanical resistance and adhesion with the substrate [29,32]. It is known that mortars that have low water retention power have a deficiency in promoting the hydration of cement in cementitious mortars. In the case of activated alkali mortars, the same behavior can be affirmed. Indeed, the loss of water is related to the loss of activating solution, although in the case of this type of material, the reaction occurs more quickly, reducing the problem related to the excessive loss of water [33,34]. In both cases, the mortar has low resistance parameters due to deficiency in the hydration reaction or in the alkaline activation reaction.

In the case of the opposite behavior, that is, in mortars with a high-water retention value, there is a deficiency in the initial adhesion between the mortar and the substrate on which the material is applied. It is known that the adhesion between mortars and substrates occurs due to three different mechanisms: physical, chemical, and micro-anchoring. Among these three mechanisms, the initial adhesion occurs due to both physical and micro-anchoring, which consists in the creation of microstructural adhesion points between the mortar and the substrate, either ceramic or concrete. Due to the mortar high water absorption, ettringite crystals are formed, in the case of cementitious mortars [29,32], or N-A-S-H (sodium aluminosilicate hydrated) and C-A-S-H gels in the case of activated alkali mortars [33,35,36]. However, if the mortar shows high water retention, even if the substrate has high absorption values, there is a difficulty in the migration of water from the mortar to the substrate, impairing micro-anchoring and consequently the initial adhesion between the materials. Other properties related to water retention are the moisture absorption of the mortar, mainly due to capillary, and the durability of the material in the hardened state due to the attack of chlorides and sulfates from the environment that can occur in mortars with poor water retention [18].

Thus, it appears that the water retention values need to be analyzed within tolerable limits that are not described by the technical standards. As recommended by some authors, a retention value between 75 and 95% should be used so that neither the mechanical properties nor the initial adhesion are impaired [24,36]. Regarding the workability properties, there are different techniques to measure the capacity of mortars to be handled or manipulated. The most applied technique is by spreading the mortar on the consistency table (flow table), but rheological techniques such as, for example, squeeze flow, dropping ball, or Vane test are also issued [37]. The main differences between the parameters obtained in these tests are summarized in Table 1.

**Table 1:** Tests to obtain mortar workability parameters.

Test	Standard	Parameters Obtained	Rheological Property
Flow Table	NBR 13276 (ABNT, 2016) [38]	Consistency index	Dynamic viscosity
Squeeze flow	NBR 15839 (ABNT, 2010) [39]	Load x displacement	Dynamic viscosity and yield stress
Dropping Ball	BS 4551 (BS, 2005) [40]	Penetration index	Yield stress
Vane Test	D4648 (ASTM, 2000) [41]	Torque x angular speed	Dynamic viscosity and yield stress

As shown in Table 1, workability tests are linked to two important rheological properties: dynamic viscosity and yield stress. It is important to understand these two concepts studied in rheology, which are the flow and deformation of matter in its plastic or liquid state, in order to evaluate the ratio between applied stress and shear force as well as the deformation in a given period of time [42]. The rheological study has applications in the study of foods, such as gelatins and vitamins, in the study of oil wells, in the evaluation of the drilling speed and extraction of petroleum materials, and in several other areas, such as the dimensioning of gas pipelines, for example [43].

Specifically in cementitious and activated alkali building materials, such as mortars, the study of rheology helps to understand the workability properties [44]. In the particular case of activated alkali materials, there is a major problem related to this subject. As the materials have a high viscosity and consequently poor workability parameters, it is sometimes necessary to use superplasticizers to correct this property [45]. However, there is a great difficulty in studying the rheology and workability of construction materials, as the applied techniques do not lead to precise values, presenting a great dispersion of data.

In addition, although the techniques detailed in Table 1 provide a notion of the dynamic viscosity (η) and the yield stress (τ_o_) of mortars, there are no reports in the literature of an accurate and reliable calculation of η and τ_o_ for alkali-activated mortars by BFS [46,47]. Although researchers report the influence of the geopolymer’s molarity on the viscosity of the activating solution [48], there are no reports on how to obtain the dynamic viscosity and flow stress of the material in its fresh state as a whole, that is, after mixing the activator with the precursor. Even though it is important to know the viscosity of the activating solution [49], it is even more important to know the rheological parameters of the material in its fresh state, as this will be the material used in civil construction operations, such as performing repairs or structures. This gap is one of the loopholes expected to be answered by the present study: What are the real rheological parameters of an alkali mortar activated by blast furnace slag (BFS) for application in structural functions?

Thus, the objective of this work is to evaluate the rheological properties and the fresh state of alkali-activated mortars using BFS and varying the sodium content of the activating solution.

## 2. Materials and Methods

**Figure 1:**
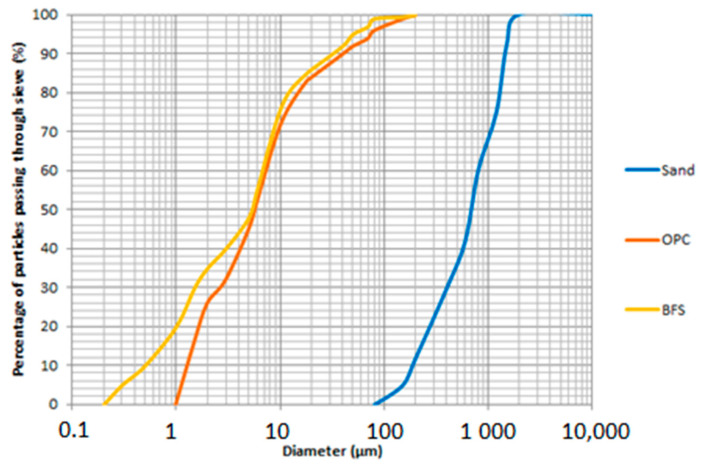
Particle size distribution of the materials used: Blast Furnace Slag (BFS); Ordinary Portland Cement (OPC) and sand.

The materials used in this research were BFS, extracted from a steel industry located in Brazil. The material comes from the production of pig iron in blast furnaces, being formed by the fusion of iron ore impurities, together with the addition of fluxes (limestone and dolomite) and coke ashes. The BFS formed, still in the molten phase, is driven by insolubility and, due to its lower density, through channels to the cooling place. At this point, the material is abruptly cooled by means of high-pressure water jets. If there is not enough time for crystals to form, the BFS granules in the amorphous glazing. The BFS used has a chemical composition of 47.49% CaO, 33% SiO_2_, 9.69% Al_2_O_3_, and 5.96 %MgO, as major compounds. The density of 1.27 g/cm³, vitrification degree of 98%, resulting in high amorphism as well as Blaine fineness of 4520 cm²/g are the main characteristics of the BFS. Figure 1 illustrates the particle size distribution of the materials used in this research.

The other materials used are commercial micropearl sodium hydroxide with 99% purity and Blaine fineness of 8000 cm²/g, potable distilled water, and washed river sand extracted from Brazil. In addition, ordinary Portland cement (OPC) was used to produce the reference mortar with 0% BFS. The sand used is passed through the 200-mesh sieve, with a fineness module of 2.25 and a maximum characteristic dimension of 1.2 mm. The OPC used presents fire loss parameters of 3.97% Fe_2_O_3_, 63.33% CaO, 19.19% SiO_2_, 5.15% Al_2_O_3_, 2.82% SO_2_, 0.92% MgO, and 4.62% of other components. In addition, it has a Blaine fineness of 4100 cm²/g.

Mortars were produced in the mixture ratio of 1:2:0.45 (binder:sand:water), using the mass amount shown in Table 2. This composition was chosen after preliminary studies carried out by Marvila et al. [50], where the authors defined the best dosing methodology for alkali-activated mortars by BFS, evaluating properties of the hardened state after 7 days of curing. In the reference composition containing cement, only OPC was considered as a binder. In activated alkaline compositions, the sum of the mass of BFS and sodium hydroxide is considered as a binder, according to the methodology defined by other authors [51,52,53]. The sodium content present in these compositions was varied in the range of 2.5 to 15% and was determined using stoichiometric calculations, as performed by Neto et al. [52,53].

The alkaline solution was produced using a Solab magnetic mixer model SL-91/A, Piracicaba, Brazil, at least 24 h in advance of the tests. The mortar masses were produced by mixing the components for 90 s in the aforementioned mixer for mortars, according to the recommendations of the Brazilian standard NBR 13276 [38].

Subsequently, rheology and property in the fresh state tests were performed. In all the tests described below, three mortar repetitions were performed for each composition. The consistency index test by the standard [38] was performed using a conical trunk shape of 12.5 cm in base diameter, 8 cm in top diameter, 6.5 cm in height, and a circular table of 50 cm in diameter and 12.0 kg in weight (consistency table SOLOTEST, São Paulo, Brazil), which applies 30 times the drop of a total height of 14 mm to the mortar. With the application of impacts, the mortar will spread on the consistency table. After the strokes were finished, the spread of the mortar on the table in three different positions was measured. The consistency index is the average among the three measured spreads.

The squeeze flow by rheological test by the Brazilian standard [39] consists of simulating the flow conditions of the mortar in the fluid state on the performance of loads. In the test, the mortar in its fresh state was applied over a cylindrical mold of 100 mm in diameter and 10 mm in height, which will be on top of a metal base of 180 mm in diameter. After molding and scraping the mortar, which was compacted with 30 strokes of a standard socket, the cylindrical mold was removed so that the mortar is not confined during the test. The mortar was compressed by a top plate with a diameter of 100 mm, attached to a load note S (30 kN of capacity) in a universal EMIC testing machine. The load was applied at a rate of 0.1 mm/s. The test was carried out until the force applied to the mortar was 1000 N or until the total displacement reached by the mortar was 9 mm. The test results were plotted on a load x displacement graph.

The dropping ball, used in a rheological test by the Brazilian standard [40], consists of releasing a standard ball in free fall on the mortar, without rotation, with a standardized drop height. The mortar was molded on the test vessel, with a diameter of 100 mm and a height of 25 mm, totaling a volume of 490.87 cm³ using a SOLOTEST equipment with an accuracy of 0.01 mm. The penetration rate of the ball in the mortar was measured and related to the workability of the mortars.

The mass density test in the fresh state and with incorporated air was carried out according to the procedure reported by Erofeev et al. [1]. The mortar was molded in a cylindrical container with known mass and volumes, in three layers, with 20 strokes applied along the perimeter of the material in each layer. After the strokes are executed, three drops are applied to the consistency table to fill the voids in the material. The density calculation consists of dividing the mass by the volume of the container. The content of incorporated air was measured with the aid of a SOLOTEST equipment with an accuracy of 0.01%, consisting of a pressure gauge of approximately 1 liter, air pump valves, and reading pressure gauge, which allow the mortar’s incorporated air to be obtained directly.

The water retention test was carried out following the Brazilian standard [54]. To perform the test, a vacuum pump is required to apply suction to the mortar and a modified Buchner funnel with 20 cm in diameter, in addition to a mercury manometer type “U” tubo, to control the suction applied in the mortar. The test consists of suctioning the mortar with a controlled pressure of 51 mm of mercury and verifying the amount of water retained by the mortar after 15 min of testing.

Afterwards, the properties of the hardened state were evaluated. The mortars were molded in cylindrical specimens 10 cm high and 5 cm in diameter applying 25 strokes to compact the material with the consistency table. For each composition and for each property evaluated, 3 samples units were used. A thermal curing procedure was carried out at 65 °C for 28 days. Compression tests were performed as per NBR 5739 [55] using a SOLOTEST hydraulic press with a capacity of 100 kN. Water absorption tests by immersion were carried out using the NBR 9778 [56]. The best composition was evaluated through complementary analysis using X-ray diffraction (XRD) performed with a Rigaku X-ray diffractometer model MiniFlex 600, 2θ scanning ranging from 0° to 90°. Morphological analyzes were also performed by scanning electron microscopy (SEM) in a Jeol microscope model JSM 6460 LV (JOEL, Tokyo, Japan).

## 3. Results and Discussion

### 3.1. Rheological Behavior

**Figure 2:**
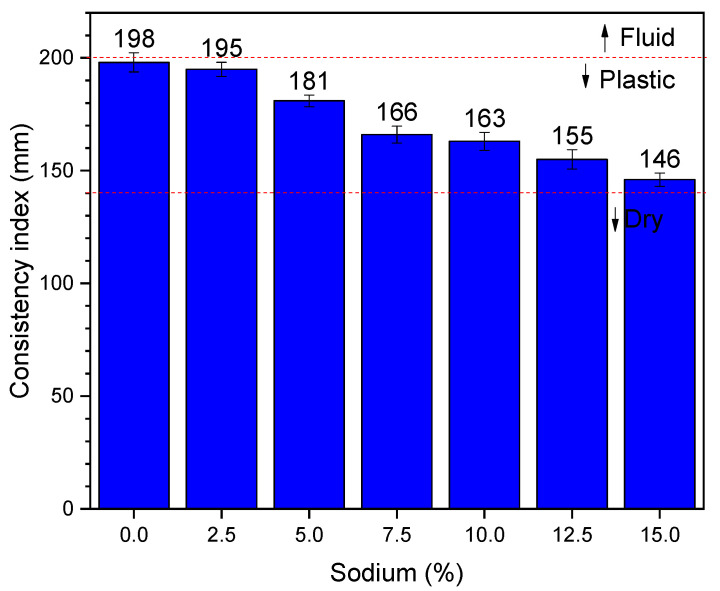
Consistency index results.

Figure 2 shows the results of the consistency index. As seen in this figure, there is a reduction in the consistency index as the sodium content increases. However, it does not show a linear trend. For example, mortar with 2.5% sodium has a consistency index of 195 mm, while mortar with 5.0% sodium has a consistency of 181 mm. This represents a drop of 14 mm in the spreading of the mortar. The same occurs with mortar containing 7.5% sodium, spreading 166 mm, which is a drop of 15 mm in relation to the composition of 5.0% sodium. This information suggests a linear trend. However, when evaluating the mortar with 10% sodium, it appears that the spread was 163 mm, which is a reduction of only 3 mm when compared to the previous composition. This pattern suggests that some relevant change occurs in the rheological characteristics of the mortar to the point that there is a change in the spreading trend between the compositions of 7.5% and 10.0% sodium. Complementing the analysis, it is observed that the mortar with 12.5% sodium has a spread of 155 mm, which is 8 mm less than the previous mortar. Moreover, the composition with 15.0% sodium has a spread of 146 mm, representing 9 mm less than the previous one. It is notable that there is a practically linear behavior between compositions 2.5–7.5% sodium and between compositions 10–15% sodium, with an important change in behavior occurring between 7.5 and 10% sodium. It is known that the spreading property is related to the dynamic viscosity of the material, indicating that a rheological change in this property occurs between the highlighted compositions [57].

Another important issue related to Figure 2 concerns the fluidity of the mortar. As reported by other authors [58,59], mortars with structural functions are considered dry when the consistency is less than 140 mm. No presently investigated mortar exhibited this behavior. In the same way, structural mortars are considered to be fluid when the spreading is greater than 200 mm, but no composition in Figure 2 shows this level of consistency. All the compositions evaluated show a consistency index between 200 and 140 mm, indicating a plastic behavior, which is ideal for application in structural functions. Evaluating the reference mortar (0%), which represents the cementitious composition, there is a reduction in workability for alkali-activated compositions. This is associated with the type of liquid used as an activator for the binders. While Portland cement is activated with water, BFS compositions are activated with alkaline hydroxide solutions. The higher viscosity of alkaline solutions is undeniable, which therefore reduces the workability of the material [60].

The workability differences observed by the alkali-activated compositions could be attributed to non-compatibility between the materials used in the manufacture of the mortar, that is, BSF, sodium hydroxide, sand, and water. However, the results obtained in the hardened state, which will be highlighted in Section 3.3, demonstrate that the alkali-activated compositions presented a mechanical performance superior to cementitious mortar, presenting the formation of tobermorite phases [56]. This proves the compatibility between the materials used in the alkali-activated mortars and validates the discussions held about the properties of the fresh state.

Comparing the results with other authors, it is observed that the results are cohesive. Alonso et al. [58] obtained consistency between 140 and 160 mm using alkali-actived mortars with the ratio 1:2 (binder:sand), varying the content of solids in the mortar. Li et al. [61] obtained results of consistency between 150 and 185 mm, using mortars based on fly ash and BFS. Rajaei et al. [59] obtained similar results, indicating that the results obtained in Figure 2 with a consistency index between 146 and 195 mm are consistent.

**Figure 3:**
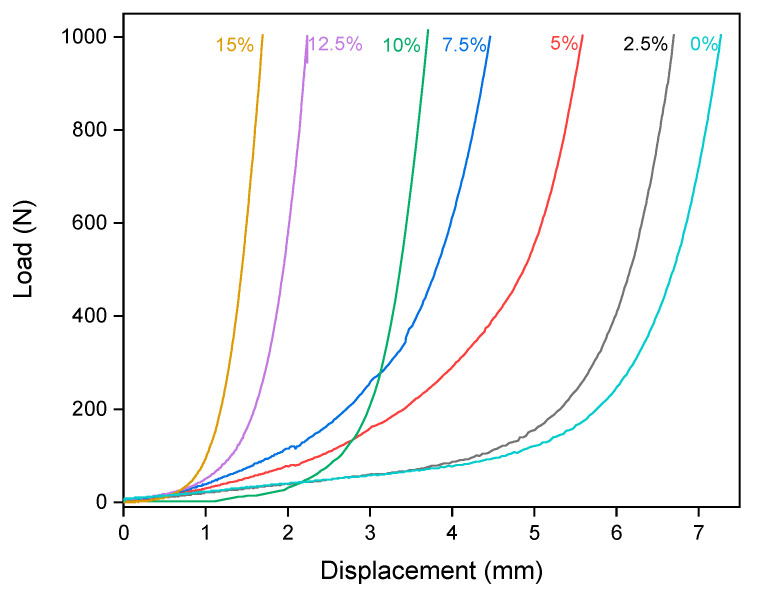
Squeeze flow results for different mixtures.

Figure 3 shows the results of squeeze flow relating the load vs. displacement curves for different sodium percentages. Through this figure, it is possible to check the information defined by the consistency test presented in Figure 2, since there is clearly a difference in behavior between the curves of 15 and 10% and between the curves of 7.5 and 0%. In the squeeze flow curve, the initial range of mortars, with an almost horizontal tendency, is related to the initial flow stress of the material, while the slope of the curves is an indication of the dynamic viscosity of the mortar. It is observed that mortars 0%, 2.5%, 5%, and 7.5% have parallel slopes to each other, showing that although in absolute values the viscosity of these mortars is different, they present the same mathematical regime of behavior. On the other hand, mortars with 10%, 12.5%, and 15% have more inclined curves, and they are also parallel to each other, proving that these mortars also follow the same mathematical model.

Based on this information, and knowing that the 0% mortar is cementitious mortar, with a Bingham fluid behavior, as highlighted in several studies [18,62,63,64], it is possible to identify that mortars with lower sodium levels (up to 7.5% in this work) also behave as a Bingham fluid, as shown by Equation (1):

(1) $$\tau =\tau_{0}+\eta \times \gamma$$

where:

*τ* = shear stress;
*τ*_0_ = initial yield stress;
*η* = dynamic viscosity;
*γ* = flow rate.

By Equation (1), it is observed that these mortars (cementitious and with a sodium content of up to 7.5%), present two parameters of relevance when evaluated from the rheological point of view. The first parameter is the initial yield stress (*τ_0_*), which is the resistance needed to remove the fluid from the inertia and start the flow, differentiating Newtonian fluids from non-Newtonian fluids. Based on the initial displacement of the curves in Figure 3, it appears that as sodium is added to the compositions, the initial flow stress increases. This indicates that the force required to remove the mortar from inertia is greater the higher the sodium content of the material. Another relevant rheological feature is the dynamic viscosity (*η*), which consists of the internal friction of fluids due to intermolecular interactions. As already discussed based on the results of Figure 2, the dynamic viscosity of mortars increased with increasing sodium content. The curve observed for mortars containing 2.5, 5, and 7.5% sodium are parallel to the 0% curve and present a very similar behavior. The curve is also close to that obtained by other authors who studied cementitious materials [21,65]. The regression of these curves is shown in Table 3. It can be seen that the mortars of 0–7.5% of sodium present a quadratic adjustment model with greater regression than the cubic model. However, mortars with sodium content of 10 to 15% have the highest R² model, which is associated with a cubic adjustment, a polynomial degree above the compositions of 0–7.5%.

This information described in Table 3 shows that the behavior of the rheological models of mortars with sodium content between 10 and 15% is different from cementitious mortars. It is worth noting that the polynomial degree of best fit is one degree higher than that of Bingham fluids. This evidences the hypothesis that these mortars behave similar to a Herschel–Bulkley fluid according to Equation (2).

(2) $$\tau =\tau_{0}+\eta \times \gamma^{n}$$

where *n* = dimensionless exponent, which in the case of these mortars is equal to 2.

The use of the dimensionless exponent equal to 2 is validated by the regression models in Table 3, where it was possible to prove that the regression of mortars with sodium content between 10 and 15% is one degree above the cementitious composition. The Herschel–Bulkley’s model is one degree above Bingham’s model, which is consistent with the results obtained [66]. In addition, it is observed that the affected term is precisely what is related to dynamic viscosity, which was modified by the use of higher sodium levels, as shown in Figure 2. In this way, the results of the consistency and squeeze flow tests are coherent.

**Figure 4:**
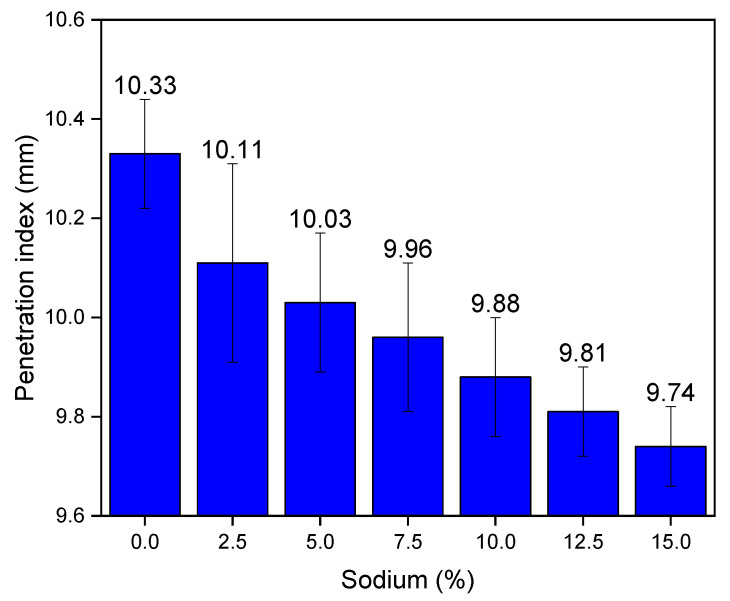
Dropping ball results.

Figure 4 presents the results of the dropping ball penetration index. It shows that the penetration index decreases as the sodium content of the alkali-activated compositions increased. However, this decrease in the penetration rate was uniform, reducing between 0.07 and 0.08 mm for every 2.5% of sodium used. As the penetration index is directly related to the initial yield stress, it appears that the use of sodium in the mortar reduces this property in a linear manner. This indicates that the changes in the mathematical models observed in Table 3 are related to the variations in dynamic viscosity and not to the initial yield stress. This information validates the information that mortars with 10 to 15% sodium content behave similar to Herschel–Bulkley fluids, while cement and sodium compositions between 2.5 and 7.5% sodium behave similar to Bingham fluids. Comparing the results obtained by [62], the authors’ penetration index values varied between 10.23 and 9.12 mm. Then, the results obtained are considered consistent.

### 3.2. Fresh State Properties

**Figure 5:**
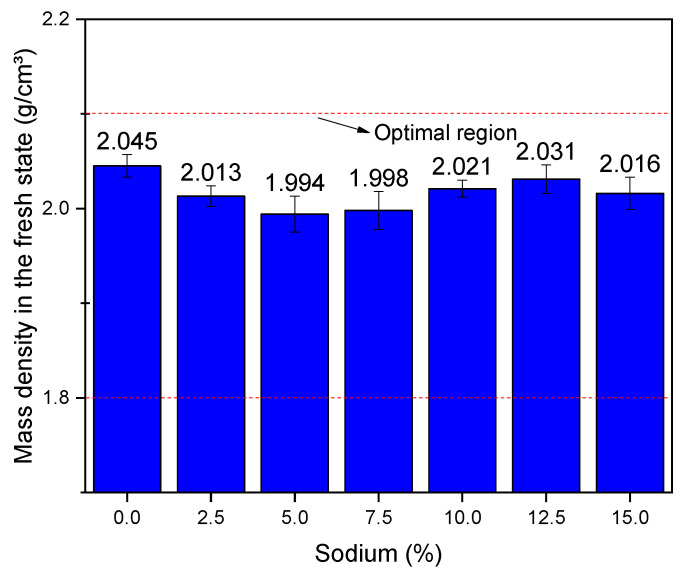
Results of mass density in the fresh state as function of the sodium content.

Figure 5 shows the results of mass density in the fresh state. Through the figure, it is not possible to observe any clear pattern in the density values, suggesting that the sodium content does not interfere in this property. In addition, it appears that the evaluated density values are within the optimal region between 1.80 and 2.10 g/cm³, as suggested by other authors [19,22]. The density results obtained are apparently similar and have no statistical difference, demonstrating that the alkali mortars activated by BFS are neither heavier nor lighter than the cement mortar in the fresh state.

**Figure 6:**
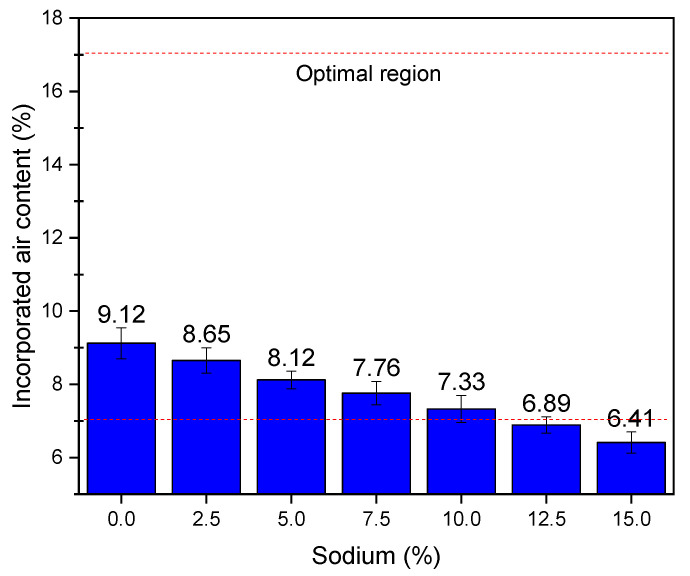
Results of incorporated air content as a function of the sodium content.

Figure 6 shows the results of incorporated air content. It is observed that there was a reduction in the content of incorporated air as the sodium content in the compositions increased. This reduction in the content of incorporated air is accompanied by a reduction in the workability of mortars, as previously reported [41]. This reduction can also be attributed to a change in rheological behavior, where mortars stop behaving with Bingham fluids and start to act as Herschel–Bulkley fluids. An explanation for the reduction of incorporated air is the fact that sodium occupies the interstices of alkali-activated mortars [67,68]. Thus, the use of larger amounts of sodium reduces the space available for the incorporated air, compromising the workability of mortars. Comparing the cementitious mortar with the mortar that contains 15% sodium, it is observed that there is a difference of 2.71% in the content of incorporated air. This difference will modify the water absorption properties, as further discussed in Section 3.3.

As for the optimum levels of incorporated air, some authors suggest the use of this parameters between 7 and 17% [18,69]. It aims that the mortars do not present high values of incorporated air. As such, there is no compromise on the mechanical strength nor that there will be very low levels of incorporated air, and a compromise on the workability properties does not occur. In this sense, the compositions 12.5% and 15% sodium do not meet the limits studied and thus should be discouraged as an application for structural functions.

**Figure 7:**
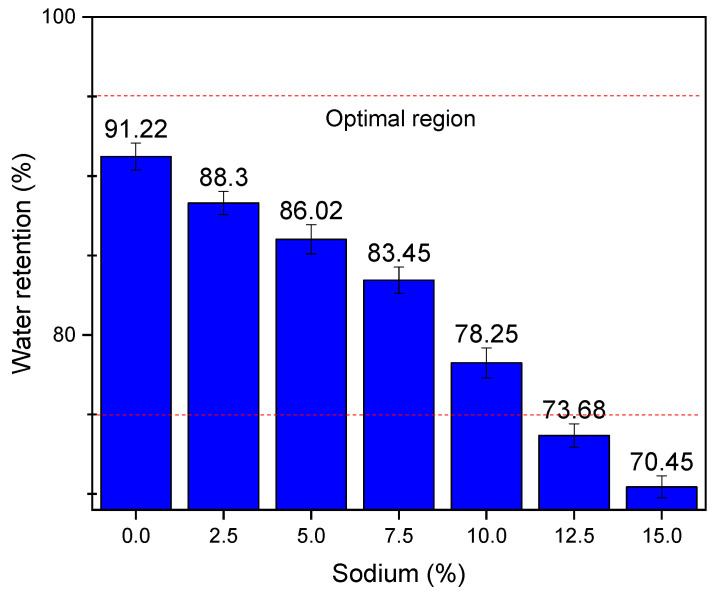
Results of water retention as a function of the sodium content.

Figure 7 shows the results of water retention. A reduction in water retention is observed as the sodium content in the activated alkali compositions increases. This is related to the interstices being occupied by sodium ions, creating a difficulty in the ability of these mortars to retain water. Regarding the limits, it is observed that no composition had more than 95% water retention; however, it is observed that the compositions of 12.5% and 15% presented retention values below 75%, as recommended by some authors [18,28]. Therefore, these two mortar compositions should be discouraged.

### 3.3. Hardened State Properties

The main objective of this article is to evaluate the rheological properties (3.1) and the fresh state (3.2) of activated alkali mortars. However, there is no point in studying mortars that have good properties in the fresh state but that do not have good parameters in the hardened state. For this reason, Table 4 presents the properties of compressive strength at 28 days and water absorption of the mortars studied. It is observed that all activated alkali mortars showed superior compressive strength than cementitious mortar and have water absorption lower than the reference mortar. The 10% sodium composition has a compressive strength of 41.07 MPa, which is approximately 2.5 times greater than the cementitious composition with a strength of 16.25 MPa.

It is also possible to reveal that the use of higher levels of sodium in mortars reduced the water absorption, which varied from 6.95% for the cementitious composition to 6.15% for the composition containing 15% sodium. One explanation for this may be related to the content of incorporated air. Indeed, where it was observed that compositions with a higher amount of sodium had a lower content of incorporated air, thus presenting less voids and less water absorption.

**Figure 8:**
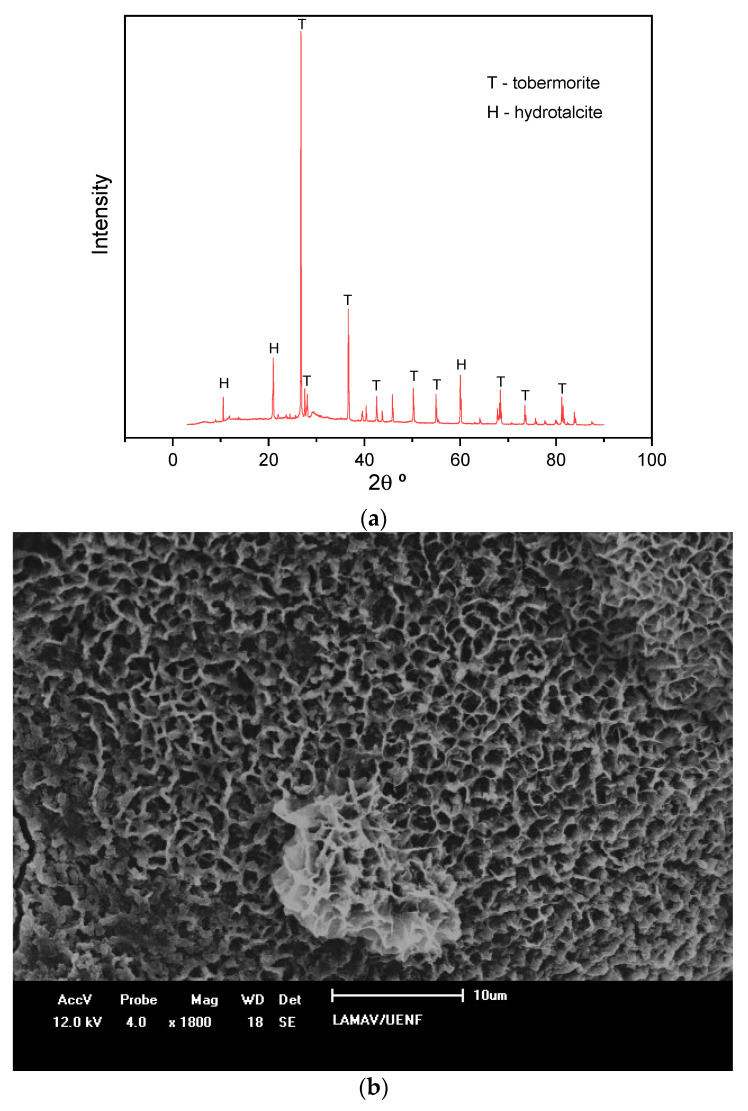
(**a**) XRD; (**b**) SEM of the composition with 10% sodium.

This happens due to the formation of tobermorite phases (C-A-S-H) in alkali-activated compositions that are much stronger than products formed with cement hydration. Figure 8 shows the results of XRD and an SEM image of the composition with 10% sodium, which displays the best mechanical performance. The formation of tobermorite is observed in the XRD, which is also detectable in the microscopic analysis illustrated by the SEM. 

It is worth noting that the alkali-activated mortars are compatible with other building materials, as reported by other authors who evaluated the possibility of these materials to be applied as tiles [2,68] and as a substitute for cementitious materials [70,71]. This proves that the alkali-activated mortars are compatible with the ceramic and cementitious materials used in civil construction. 

In addition, the durability of these mortars is superior to cementitious materials, as indicated in the literature [69]. This happens due to the formation of tobermorite (C-A-S-H), which presents network cross-connections, providing the mortars with superior performance as compared with hydrated cement with C-S-H networks [4,72]. Some authors have already proved that alkali-activated mortars perform better in conditions of saturation, such as rain and fire [2,73]. The same is true in air pollution conditions [74]. Therefore, the alkali-activated compositions herein evaluated are relevant for civil construction applications, presenting properties superior to the cementitious composition and emphasizing the importance of the present results.

## 4. Conclusions

The properties of the fresh and rheological state of alkali-activated mortar by means of blast furnace slag (BFS) were evaluated. It was observed that mortars containing between 2.5 and 7.5% of sodium show the same rheological behavior as cementitious mortars. That is, they meet the Bingham fluid model, which is confirmed by the results of consistency and squeeze flow. However, mortars containing between 10 and 15% sodium act similar to Herschel–Bulkley fluids (H-B model), where the dynamic viscosity properties become more significant in the fluid’s behavior. This explains the pattern observed by the consistency index, with a linear reduction between 2.5 and 7.5% of sodium and with another reduction range between 10 and 15% of Na_2_O. It is noteworthy that the consistency index results have a direct association with the dynamic viscosity of the material.

In the case of the dropping ball test results, related to the initial flow tension, a linear reduction was observed, between 0.07 and 0.08 mm for each 2.5% of sodium used. This indicates that the use of larger amounts of sodium in the alkaline solution reduces the two main rheological properties of alkali-activated mortars, but in a different way. The initial flow stress, calculated by the dropping ball measures, is uniformly reduced the higher the %Na_2_O. However, the dynamic viscosity reduces in two different regimes: similar to the cementitious composition, between 2.5 and 7.5% sodium, and in a more intense regime between 10 and 15% sodium. The mathematical models of regression of the squeeze flow curves prove that the compositions between 2.5 and 7.5% present a more adjusted R² for a quadratic model, similar to the 0% composition. In the case of compositions containing 10 to 15% sodium, the best regression model is a cubic, H-B model, which is a polynomial degree above the Bingham model. This helps to conclude about the rheological models presented by alkali-activated mortars, as mentioned above.

The mass density properties in the fresh state did not vary, but the water retention properties and voids index suffered a great reduction. Moreover, the use of compositions containing 12.5% and 15% sodium is not recommended, since the water retention of these compositions was less than 75% and the content of incorporated air was less than 7%. The reduction of these properties is attributed to the presence of sodium ions in the interstices of the material, which reduce the space for the incorporated air and were a barrier that prevents water retention. Thus, it is concluded that the techniques used in this research made it possible to achieve the proposed objective, of characterizing alkali-activated mortars with compatible properties as civil construction materials.

## Figures and Tables

**Table 2:** Compositions of mortars.

%Na_2_O	OPC (g)	BFS (g)	Sand (g)	Sodium Hydroxide (g)	Water (g)
0.0%	300.00	0.00	600.00	0.00	135.00
2.5%	0.00	281.10	580.93	9.37	128.60
5.0%	0.00	272.30	582.16	18.78	126.76
7.5%	0.00	263.47	583.39	28.23	124.91
10.0%	0.00	254.60	584.63	37.72	123.06
12.5%	0.00	245.69	585.87	47.25	121.19
15.0%	0.00	236.74	587.12	56.82	119.32

**Table 3:** Regression of the squeeze flow curves.

Composition	Quadratic Regression		Cubic Regression	
0% (OPC)	y = 24.95x² − 87.66x + 49.50	R² = 0.97	y = 8.78x³ − 64.90x² + 129.45x − 21.16	R² = 0.90
2.5%	y = 26.66x² − 96.67x + 47.44	R² = 0.98	y = 11.15x³ − 75.71x² + 138.95x − 24.64	R² = 0.91
5.0%	y = 38.16x² − 71.67x + 32.79	R² = 0.97	y = 11.13x³ − 49.60x² + 94.61x − 11.81	R² = 0.95
7.5%	y = 61.64x² − 93.21x + 34.83	R² = 0.97	y = 22.32x³ − 78.36x² + 118.96x − 12.87	R² = 0.95
10.0%	y = 112.62x² − 247.01x + 66.45	R² = 0.85	y = 66.55x³ − 256.38x² + 243.32x − 29.78	R² = 0.97
12.5%	y = 318.38x² − 354.66x + 65.29	R² = 0.86	y = 237.60x³ − 451.71x² + 257.63x − 17.07	R² = 0.99
15.0%	y = 578.10x² − 496.42x + 62.22	R² = 0.88	y = 496.38x³ − 628.24x² + 238.19x − 16.03	R² = 0.99

**Table 4:** Properties of mortars.

%Na_2_O	Compressive Strength (MPa)	Water Absorption (%)
0.0%	16.25 ± 0.74	6.95 ± 0.15
2.5%	20.25 ± 0.85	6.58 ± 0.17
5.0%	27.00 ± 0.34	6.52 ± 0.21
7.5%	37.25 ± 0.53	6.47 ± 0.16
10.0%	41.07 ± 0.62	6.32 ± 0.15
12.5%	35.00 ± 0.42	6.21 ± 0.19
15.0%	27.41 ± 0.41	6.15 ± 0.25

## Data Availability

Not applicable.
